# Supplementary material for: Graphene Based Waveguide Polarizers: In-Depth Physical Analysis and Relevant Parameters
Source: Sci Rep. 2015 Nov 19;5:16949. doi: 10.1038/srep16949 (PMC4652265; doi:10.1038/srep16949)
Supplement: Supplementary Information [file srep16949-s1.pdf]

Supplementary Information

Graphene Based Waveguide Polarizers: In-Depth Physical Analysis and Relevant Parameters

Rafael E. P. de Oliveira* and Christiano J. S. de Matos

MackGraphe – Graphene and Nanomaterials Research Center, Mackenzie Presbyterian University, São Paulo, 01302-907, Brazil
*rafael.oliveira1@mackenzie.br

1. Polarization Extinction Ratio Dependence on Graphene's Fermi level for Different Waveguides

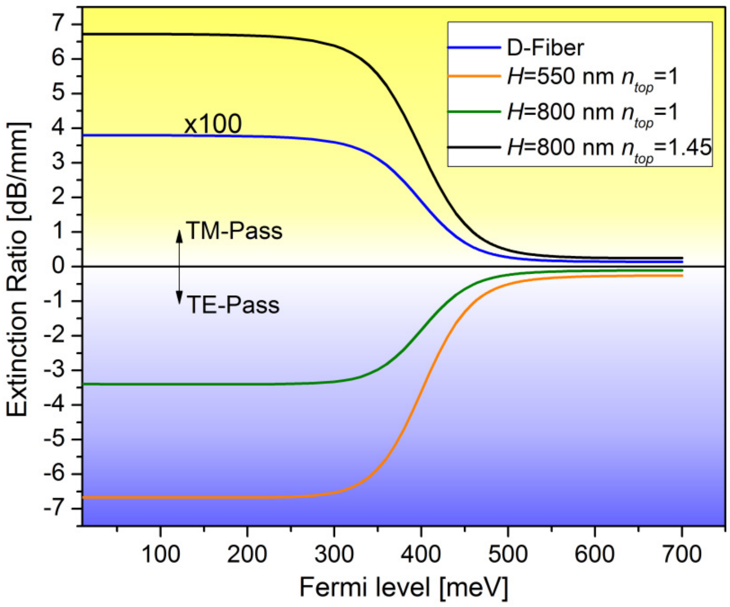

Figure S1. Polarization extinction ratio for the silicon nitride waveguide described in the main text, with different parameters (Height, *H*, and superstrate refractive index, n_{top}) and for the D-shaped optical fiber reported by *Lee et al.*[14]. The D-Fiber extinction ratio was multiplied by 100 due to the lower interaction between light and graphene in this geometry compared to the interaction in integrated waveguides. There are no transitions between TE and TM pass polarization as a function of the Fermi level for any case.

2. Field and Intensity Distributions, and $U_{||}$ Values, for various waveguide geometries

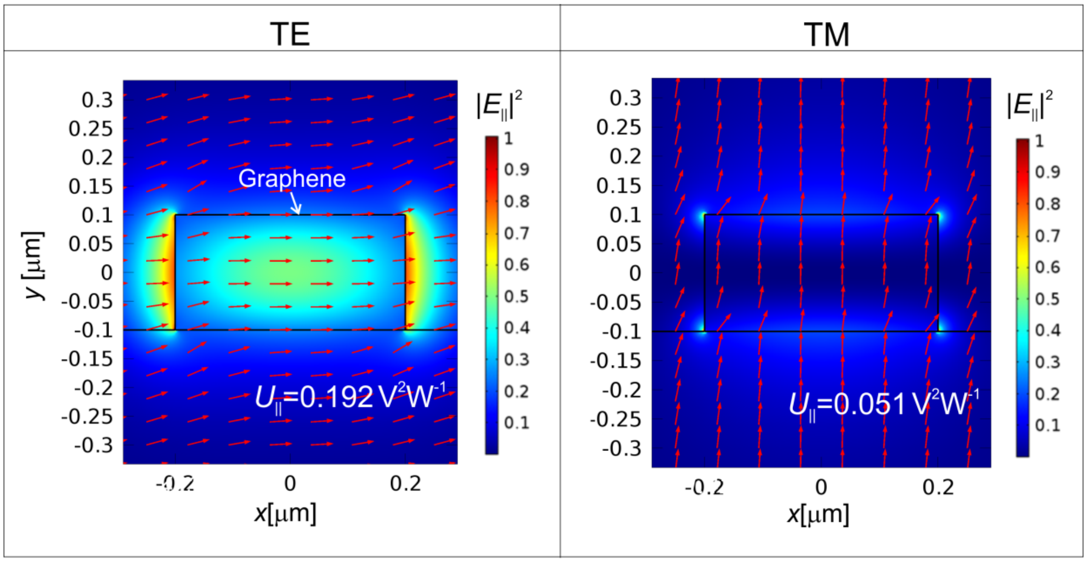

Figure S2. Parallel electric field intensity distribution $|E_{||}|^2$ for the TE and TM modes in the silicon waveguide with graphene reported by Kou *et al.*[12]. The red arrows indicate the electric field direction of each mode. The $U_{||}$ parameter value is given for each case.

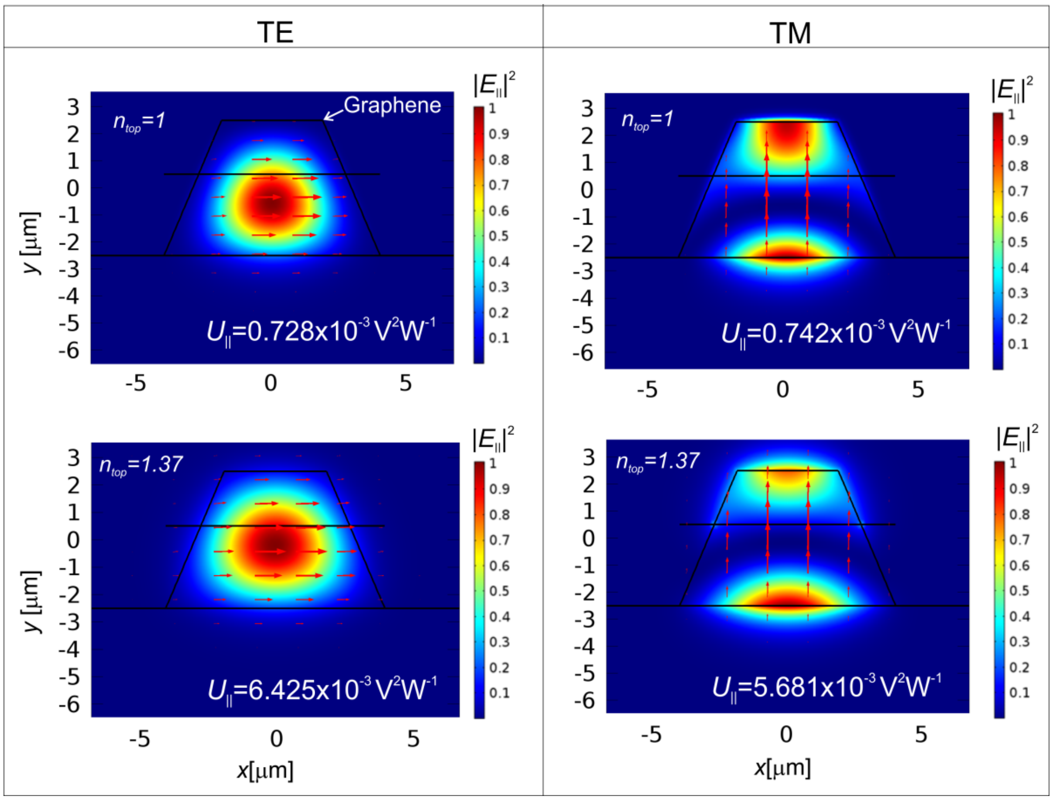

Figure S3. Parallel electric field intensity distribution $|E_{||}|^2$ for the TE and TM modes in the polymeric waveguide with graphene reported by Kim and Choi[13]. The red arrows indicate the electric field direction of each mode and the $U_{||}$ parameter is given for each case. Considering the waveguide geometry, it is expected that the graphene partially covers the sides of the waveguide, in addition to its top. The horizontal lines at y=0.5 μm indicate the points above which the core perimeter is assumed to be covered with graphene.

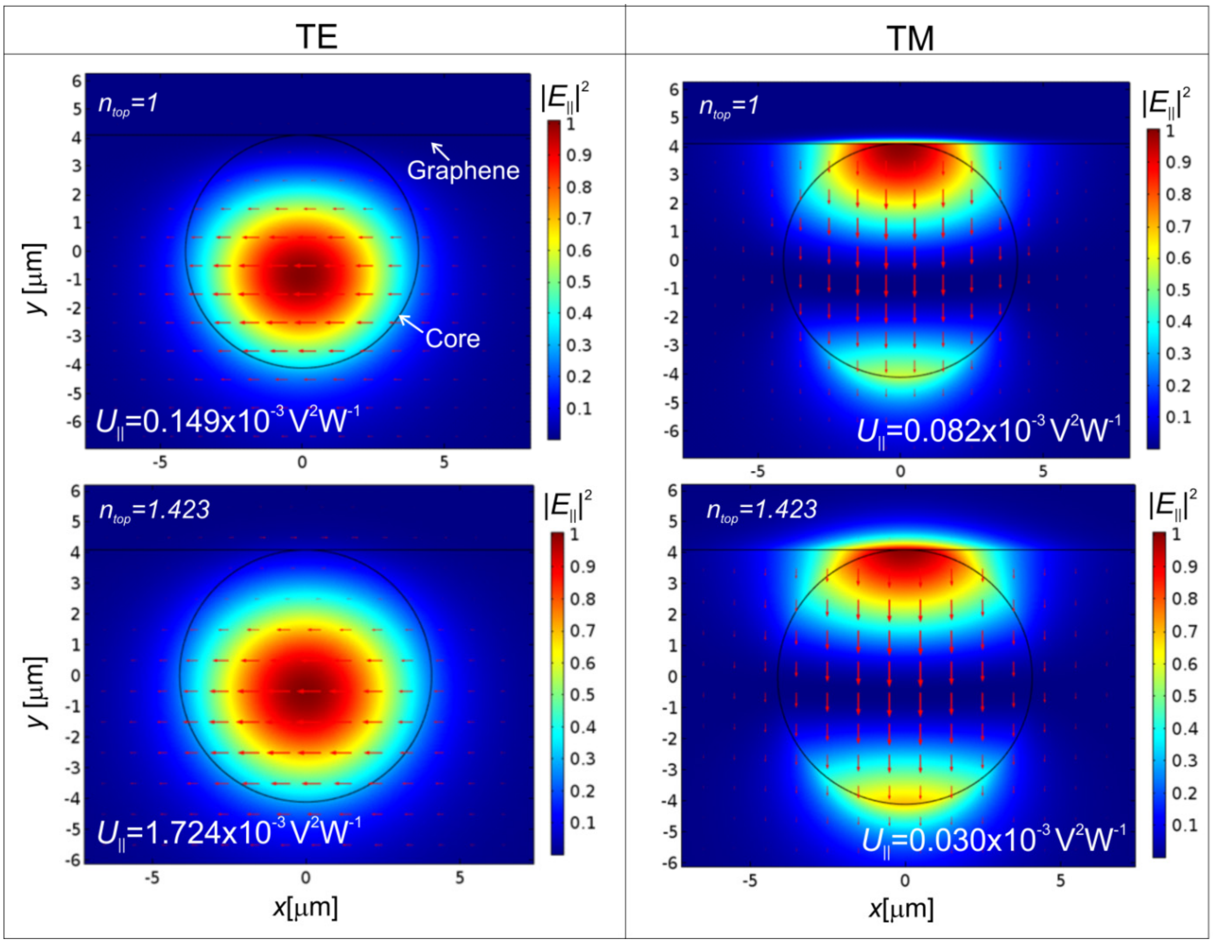

Figure S4. Parallel electric field intensity distribution $|E_{||}|^2$ for the TE and TM radiations in the D-shaped optical fiber with graphene reported by Lee *et al.*[14]. The red arrows indicate the electric field direction of each mode. Simulations were performed for superstrate refractive indices n_{top} of 1 and 1.423. The $U_{||}$ parameter is for each case. By increasing the n_{top}, light interaction with graphene is increased for the TE radiation and decreased for the TM radiation. This results in a proportional increase (decrease) of the optical absorption for the TE (TM) radiation, in agreement with the reported experimental results[14].
